# Ultrastructural Study of *Cryptococcus neoformans* Surface During Budding Events

**DOI:** 10.3389/fmicb.2021.609244

**Published:** 2021-03-01

**Authors:** Glauber R. de S. Araújo, Carolina de L. Alcantara, Noêmia Rodrigues, Wanderley de Souza, Bruno Pontes, Susana Frases

**Affiliations:** ^1^Laboratório de Ultraestrutura Celular Hertha Meyer, Instituto de Biofísica Carlos Chagas Filho, Universidade Federal do Rio de Janeiro, Rio de Janeiro, Brazil; ^2^Laboratório de Biofísica de Fungos, Instituto de Biofísica Carlos Chagas Filho, Universidade Federal do Rio de Janeiro, Rio de Janeiro, Brazil; ^3^Centro Nacional de Biologia Estrutural e Bioimagem (CENABIO), Universidade Federal do Rio de Janeiro, Rio de Janeiro, Brazil; ^4^Laboratório de Pinças Óticas (LPO-COPEA), Instituto de Ciências Biomédicas, Universidade Federal do Rio de Janeiro, Rio de Janeiro, Brazil

**Keywords:** *Cryptococcus neoformans*, polysaccharide capsule, cell wall, budding, electron microscopy

## Abstract

*Cryptococcus neoformans* is a fungal pathogen that causes life-threatening infections in immunocompromised individuals. It is surrounded by three concentric structures that separate the cell from the extracellular space: the plasma membrane, the cell wall and the polysaccharide (PS) capsule. Although several studies have revealed the chemical composition of these structures, little is known about their ultrastructural organization and remodeling during *C. neoformans* budding events. Here, by combining the latest and most accurate light and electron microscopy techniques, we describe the morphological remodeling that occurs among the capsule, cell wall and plasma membrane during budding in *C. neoformans*. Our results show that the cell wall deforms to generate a specialized region at one of the cell’s poles. This region subsequently begins to break into layers that are slightly separated from each other and with thick tips. We also observe a reorganization of the capsular PS around the specialized regions. While daughter cells present their PS fibers aligned in the direction of budding, mother cells show a similar pattern but in the opposite direction. Also, daughter cells form multilamellar membrane structures covering the continuous opening between both cells. Together, our findings provide compelling ultrastructural evidence for *C. neoformans* surface remodeling during budding, which may have important implications for future studies exploring these remodeled specialized regions as drug-targets against cryptococcosis.

## Introduction

Fungal infections that cause systemic mycoses have become a major threat, a clinical and a pharmaceutical challenge since the end of the 20th century, especially affecting individuals with an immunological impairment (Perfect, 2013). There is evidence showing that the increase in fungal infections is related to glucocorticoid therapy, immunotherapy, oncological and hematological diseases, increased number of transplants, surgical procedures, individuals living with acquired immunodeficiency syndrome (AIDS), among others (Singh et al., 2008; Henao-Martínez and Beckham, 2015; Liao et al., 2016).

*Cryptococcus* spp., of which *Cryptococcus neoformans* is the main representative of the genus, is a basidiomycete that presents itself as a haploid and spherical yeast surrounded by a polysaccharide (PS) capsule, a unique feature among eukaryotes (McFadden et al., 2006). *Cryptococcus* spp. has a global distribution and causes about 181,100 deaths per year worldwide (Park et al., 2009; Rajasingham et al., 2017). The host becomes infected after inhaling spores or desiccated yeasts (Ellis and Pfeiffer, 1990) and the infection can either take its latent form, without causing any clinical symptoms or manifest itself in the acute form of the disease (Goldman et al., 2010). Given that *Cryptococcus* spp. has a special tropism toward the central nervous system (CNS) and can colonize the CNS through many concomitant infection routes (Mitchell et al., 1995; Vu et al., 2014; Mourad and Perfect, 2018), one can consider cryptococcal meningitis as the most severe cryptococcosis scenario (Casadevall and Perfect, 2008; World Health Organization, 2018).

The success of the infection is based on the ability of the fungus to evade the host’s immune system. During its evolution, *Cryptococcus* spp. developed several adaptation mechanisms, known as virulence factors. Some examples are (i) melanin production and cell wall remodeling (resistance to cell-mediated death and immunomodulation) (Huffnagle et al., 1995; Wang et al., 1995; Doering et al., 1999; Liu et al., 1999; Gómez and Nosanchuk, 2003), (ii) production of superoxide dismutase (protection against toxic free radicals) (Cox et al., 2003), (iii) phospholipase and urease secretion (intracellular growth, diffusion and proliferation) (Cox et al., 2000, 2001), (iv) phenotypic switching (immune evasion) (Goldman et al., 1998; Fries et al., 2001), (v) cellular gigantism (immune evasion) (Okagaki et al., 2010; Zaragoza and Nielsen, 2013; Trevijano-Contador et al., 2018; Zaragoza, 2019), (vi) thermotolerance (Johnston et al., 2016; Araújo et al., 2017; Bloom et al., 2019), and (vii) PS production, which is the main virulence factor used by *C. neoformans* (Zaragoza et al., 2009; Araujo et al., 2012; Zaragoza, 2019). Most of these features are believed to have been acquired through selective pressures and are likely to be the result of interactions with environmental predators, such as amoebae and nematodes (Casadevall and Pirofski, 2007; Albuquerque et al., 2019).

After production, *Cryptococcus* spp. PS can be either secreted to the extracellular milieu through vesicles (Rodrigues et al., 2007; de Oliveira et al., 2020) or transported to the cell wall, where it forms the physical structure of the capsule *in situ*. Depending on its fate, PS acquires different physicochemical and rheological properties (Pontes and Frases, 2015; Araújo et al., 2019; Zaragoza, 2019). Due to its unique morphology and the fact that it is pivotal for the establishment of pathogenesis, the PS capsule is the most distinctive feature of the *Cryptococcus* genus. It is extremely dynamic, highly hydrated and can be modified in response to the environment (Maxson et al., 2007; Matsumoto et al., 2019; Zaragoza, 2019; Vij et al., 2020). This structure appears at the surface of the cell wall and its main roles are to protect the cell against the host’s defense factors and to interfere with immune response mechanisms (Perfect and Casadevall, 2011). To anchor to the cell wall, the PS molecules from the capsule interact with α-1,3 glucans (Reese and Doering, 2003). However, the full mechanism that dictates the interaction between the capsule and the cell wall is far from being completely understood but is supposed to involve molecular interactions, such as hydrogen bonds or other covalent and/or non-covalent interactions, between the components of both structures (Agustinho et al., 2018).

The fungal cell wall is an intricate network of macromolecules considered as the primary determinant of fungal resistance to stress and environmental aggressions. It provides not only strength and rigidity to maintain the cell conformation but also the flexibility to support morphological changes, such as cell growth and budding (Roncero, 2002; Ruiz-Herrera et al., 2002; Adams, 2004). The cryptococcal cell wall also serves as the scaffold for the assembly/anchoring of the PS capsule. The cell wall is comprised of a matrix containing glycoproteins and glucose (Glc), *N*-acetylglucosamine (GlcNAc), and glucosamine (GlcN) polymers, whose main constituents are glucans, chitin, and chitosans (Perfect and Casadevall, 2011). The glucans are divided into α- and β-glucans. A large fraction of α-glucans present α-1,3 links (James et al., 1990; Bose et al., 2003; Wang et al., 2018) whereas the majority of β-glucans are comprised by β-1,3 and β-1,6 bonds (Manners et al., 1973; James et al., 1990; Wang et al., 2018). Chitin, another constituent of the cell wall, is a water-insoluble β-1,4-GlcNAc polymer, that associates with each other to form chitooligomers (chitooligosaccharides). These chitooligomers contain between three to twenty residues of β-1,4-GlcNac, which provides the cell wall with rigidity and structural integrity under various environmental conditions. In *C. neoformans*, chitooligomers are also incorporated into the capsular network and interact with glucuronoxylomannan (GXM) to form complex glycans. Chitin-derived oligomers have also been shown to regulate capsular architecture in *C. neoformans*, playing an indirect role in cryptococcal pathogenesis (Ramos et al., 2012; Fonseca et al., 2013). Finally, these oligomers have also been detected on the outermost surface of the capsule, suggesting their potential to be recognized by host receptors, possibly affecting cryptococcal pathogenesis (Fonseca et al., 2013). Cell wall chitins can also be deacetylated to generate chitosan, a more soluble and flexible glucosamine polymer. *C. neoformans* have high levels of chitosan that can exceed chitin amounts up to 10 times (Banks et al., 2005). Cells without chitosan grow slower than the wild type and present impaired cell integrity and reduced virulence in animal models (Baker et al., 2011). Overall, glycoproteins are crucial components of the cell wall in fungi, as they act in critical processes, including signal transduction, conjugation, cell wall synthesis and iron acquisition. Glycoproteins are modified by *N*-oligosaccharide and *O*-oligosaccharide bonds, usually mannosylated structures, which syntheses are initiated in the endoplasmic reticulum and the Golgi complex (Wang et al., 2018). Glycans linked to the cryptococcal proteins contain xylose (Xyl) and Xyl-phosphate moieties (Reilly et al., 2011; Park et al., 2012; Lee et al., 2015). Even though the full spectrum of glycans has not yet been completely elucidated, it is known to contain sialic acid that plays an anti-phagocytic role and may represent a virulence factor in the initial stages of infection (Rodrigues et al., 2002).

Polarized cell growth (PCG) and directional cell division (DCD) are fundamental and essential processes for the development of eukaryotes. PCG involves asymmetric growth of a cell region to form specific cell structures or shapes. The resulting specialized structures are critical for the function of several cell types and can help mediate various cell interactions during development. Some examples are the absorption of nutrients by epithelial cell microvilli (Mooseker, 1985) and the interaction between T and B cells (Kupfer et al., 1986; Madden and Snyder, 1998). Likewise, PCG in fungi occurs by inserting new material into the plasma membrane via the secretory pathways together with concomitant cell wall remodeling. It can be either triggered by internal factors, for example progression of the cell cycle, or by external factors, such as changes in the environment or nutritional status (Bassilana et al., 2020). Although filamentous fungi and yeasts show obvious differences in their growth modes, they share three basic properties that allow for PCG and the formation of a diverse variety of cellular forms: (i) symmetry breaking, in which an initially isotropic cell generates a polarized growth axis, (ii) maintenance of polarity, which refers to the stabilization of the polarity axis so that polar growth is maintained, and finally, (iii) depolarization, in which polarity is lost in a controlled manner. The balance between polarity maintenance and depolarization generates diversity in fungal cell forms (Lin et al., 2014). Fungal cells are not always polarized during the early stages of development. They usually undergo an initial period of non-polar isotropic expansion (for example, spores from yeast stem cells). Ultimately, however, cell symmetry must be broken, and a polarity axis generated, both for the selection of the budding site or for the development of polar structures, such as hyphae (Madden and Snyder, 1998; Chant, 1999).

Many of the studies that focused on cell division events for pathogenic yeasts used paradigms established in the ascomycete *Saccharomyces cerevisiae*. However, basidiomycete yeasts, such as *C. neoformans*, show conserved and distinct features in their morphogenesis, only producing hyphae during sexual differentiation (Lin et al., 2014) and their produced spores are quite infectious and can be the primary particle inhaled during natural infection (Giles et al., 2009; Velagapudi et al., 2009). Once inhaled, upon reaching the lungs, the Cryptococci spores germinate to produce yeast cells. In the context of infection, this fungus grows within the human host almost exclusively in the form of yeasts. Histopathological studies have shown that hyphal forms are rarely found during human infections by *C. neoformans* (Baker and Haugen, 1955; Shadomy and Utz, 1966; Fu et al., 2019).

Since the last century, there has been a great interest in deciphering the chemical composition of *C. neoformans* cell surface; however, little is known about its ultrastructural organization and remodeling during important events of *C. neoformans* biology. In the present work, we combined light and electron microscopy techniques to describe the morphological remodeling that occurs within the capsule, cell wall and plasma membrane during the budding phenomenon in *C. neoformans*.

## Materials and Methods

### Microorganisms

The strain used in this work was *C. neoformans* var. *grubii* H99 (clinical isolate, kindly provided by Professor Arturo Casadevall – Johns Hopkins Bloomberg School of Public Health, Baltimore, MD, United States), a wild type strain available in the American Type Culture Collection (ATCC catalog number 208821).

### Capsule Induction and Culture Conditions

Yeasts were grown in Sabouraud Dextrose Broth (Kasvi, PR, Brazil) medium at 37°C with constant agitation at various times, depending on the experimental conditions. For video microscopy observations, the yeasts were taken directly from Sabouraud Dextrose Agar (Kasvi, PR, Brazil) and, subsequently, added in liquid culture medium and processed, as described below. To induce capsule formation, the yeasts were kept at 37°C for 7 days in a nutrient-deprived medium called Minimal Medium (MM) containing only 15 mM glucose, 10 mM MgSO_4_7⋅H_2_O, 29 mM KH_2_PO_4_, 13 mM glycine and 3 μM thiamine (all compounds from Merck Millipore, Darmstadt, Germany).

### Video Microscopy

An initial inoculum of 10^4^ cells/mL in Sabouraud medium was added to 35 mm glass-bottom dishes (Thermo Scientific^TM^ Nunc Glass-bottom Dish, Waltham, MA, United States) and observed under a Nikon Eclipse TE300 inverted microscope equipped with a CFI Achromatic LWD ADL 40× objective lens. For 5 h, phase-contrast images were captured every minute using a Hamamatsu C2400 CCD camera (Hamamatsu, Japan). Images were then mounted into stacks and analyzed using the ImageJ 1.8.0 software (NIH, Bethesda, MD, United States^1^) (AbraÌmoff et al., 2004; Schneider et al., 2012).

### Conventional Fluorescence Microscopy and Structured Illumination Microscopy

Yeast cells (10^6^) were centrifuged at 6,708 *g* for 5 min, resuspended in 4% (v/v) paraformaldehyde (Electron Microscopy Sciences, Hatfield, PA, United States) in phosphate-buffered saline (PBS) (137 mM NaCl, 2.7 mM KCl, 10 mM Na_2_HPO_4_, and 1.8 mM KH_2_PO_4_) pH 7.4 and incubated for 30 min at room temperature. Next, fixed cells were washed twice with PBS and incubated with 1% bovine serum albumin (Sigma Aldrich, Darmstadt, Germany) in PBS for 1 h at room temperature. The cells were then incubated for another hour at room temperature with 18B7 mAb (10 μg/mL). The 18B7 mAb is a mouse IgG1 with a high affinity for GXM from different cryptococcal serotypes (Goldman et al., 1998). After three washes in PBS, cells were incubated with 10 μg/mL of the anti-mouse Alexa Fluor^®^ 594 secondary antibodies (Thermo Fisher Scientific, Waltham, MA, United States) for 1 h at room temperature. Again, cells were washed with PBS buffer and incubated with Uvitex2B (Polyscience Inc., Warrington, PA, United States) for 1 h at room temperature and subsequently, vigorously washed four times with PBS buffer to remove all Uvitex2B dye to minimize background.

Cell suspensions were mounted on glass coverslips and observed using an ZEISS Axio Observer or an ZEISS Elyra PS.1 microscope (Zeiss, Germany). Images were acquired with their respective software packages (Zen Blue or Zen Black) and subsequently processed using ImageJ 1.8.0 software (NIH, Bethesda, MD, United States – See text footnote 1) (AbraÌmoff et al., 2004; Schneider et al., 2012).

### Conventional and High-Resolution Scanning Electron Microscopy

The cells of interest were washed three times in PBS pH 7.4 and fixed in 2.5% glutaraldehyde solution grade I (Electron Microscopy Sciences, Hatfield, PA, United States) in sodium cacodylate buffer 0.1 M pH 7.2 for 1 h at room temperature. Then, the cells were washed three times in 0.1 M sodium cacodylate buffer pH 7.2 containing 0.2 M sucrose and 2 mM MgCl_2_ (Merck Millipore Darmstadt, Germany), and adhered to 12 mm diameter round glass coverslips (Paul Marienfeld GmbH & Co. KG, Germany) previously coated with 0.01% poly-L-lysine (Sigma-Aldrich, Darmstadt, Germany) for 20 min. Adhered cells were then gradually dehydrated in ethanol (Merck Millipore, Darmstadt, Germany) series (30, 50, and 70% for 5 min and 95 and 100% twice for 10 min). The coverslips were then critical-point-dried using an EM DPC 300 critical point drier (Leica, Germany) and mounted on specimen stubs using a conductive carbon adhesive (Pelco Tabs^TM^, Stansted, Essex, United Kingdom).

Next, for conventional scanning electron microscopy, the samples were coated with a thin layer of gold or gold-palladium (10–15 nm) using the sputtering method (Balzers Union FL-9496, Balzers, FL) and visualized in a FEI Quanta^TM^ 250 microscope operating at 10–20 kV. The images were collected with the SmartSEM and xT microscope Server/xT microscope Control software. On the other hand, for high-resolution scanning electron microscopy, samples were sputter-coated with a 4–5-nm-thick platinum layer using a Leica EM SCD 500 sputtering device (Wetzlar, Germany) and visualized with either a FEI Magellan^TM^ or a FEI Quanta^TM^ 450 FEG (FEI Company, Oregon, United States) scanning electron microscope operating at 1–5 kV. The images were collected with the software packages of each of these microscopes.

Quantification of PS fiber anisotropy was performed using FibrilTool (Boudaoud et al., 2014), an ImageJ plug-in that determines the average orientation of a fiber array. The anisotropy value ranges from a maximum of 1, when all fibers point in the same direction, to a minimum of 0, when fibers are randomly oriented. We analyzed a collection of 23 different images of budding events. For each image, anisotropy values were obtained from SR and regions outside SR of both mother and daughter cell. We defined SR as the entire region where PS fibers were specially oriented in each cell (mother and daughter), while region outside SR was considered as the entire remaining region in each cell (see Figure 6E for more details). We chose the areas to be analyzed by manually selecting (using the plugin selection tool) the regions. Then, the values for each region (SR and region outside SR) and of each cell (mother and daughter) were obtained. As the anisotropy values for the SRs of both mother and daughter cells did not show statistically significant differences between them, we decided to join all in one data set. The same was done for the regions outside SR of both mother and daughter cells, thus generating two different data sets, represented as means ± standard errors.

### Transmission Electron Microscopy

The cells were washed three times in PBS pH 7.2 and subsequently fixed in 2.5% (v/v) glutaraldehyde solution grade I (Electron Microscopy Sciences, Hatfield, PA) in 0.1 M sodium cacodylate buffer pH 7.2 and microwaved (350 W, 3 pulses of 30 s each with an interval of 60 s between pulses) (Benchimol et al., 1993; Giberson et al., 2003). Subsequently, the cells were washed three times in 0.1 M sodium cacodylate buffer pH 7.2. The cells were then post-fixed using an adapted Osmium-Thiocarbohydrazide-Osmium (OTO) protocol, that was chosen because the use of thiocarbohydrazide (TCH) as a binding agent in the process called osmium impregnation, acting as osmophilic, enhances the osmium staining of lipid components such as cell membranes. The TCH works by attaching to the osmium already present and acting as a bridge to allow the deposition of additional osmium, as well as making the sample more conductive to electrons (Seligman et al., 1966; Murakami et al., 1983; Willingham and Rutherford, 1984; Tapia et al., 2012). However, in order to adjust to our experimental conditions, we had to perform several adaptations to the standard OTO protocol. Thus, we decided to describe in detail all the solutions used, together with their respective incubation time, as well as any extra procedures performed. Briefly, cells were incubated in a post-fixative with 1% (v/v) osmium tetroxide (OsO_4_) (Ted Pella, Inc.), 0.8% (v/v) potassium ferrocyanide (Electron Microscopy Sciences, Hatfield, PA) and 5 mM calcium chloride, in 0.1 M cacodylate buffer (pH 7.2) for 10 min, washed twice in water, and then incubated in 1% (w/v) TCH (Sigma, Darmstadt, Germany) in water, for 5 min. After three washes in water, cells were again incubated in the post-fixative osmium solution for 2 min and finally washed three times in water. Next, cells were gradually dehydrated in acetone (Merck Millipore, Darmstadt, Germany) series: 50, 70, 90, and two subsequent 100%. All the dehydration procedures were performed in the microwave (350 W, 10 s pulses for each step). Although not quite usual, the microwave stimulus has a great advantage in reducing the fixation, post-fixation and resin polymerization times, mainly because the electromagnetic waves promote an increase in kinetic energy favoring the diffusion of fixatives and other fluids, causing better preservation of the material if properly used (Benchimol et al., 1993; Giberson et al., 2003; Wendt et al., 2004). Then, the Spurr resin (Electron Microscopy Sciences, Hatfield, PA) was gradually used to substitute acetone in the following proportions acetone: Spurr (v:v): 3:1, 2:1, 1:1, 1:2, 1:3 and finally pure Spurr. Each mixture was also submitted to the same microwave cycles for 2 min, except the last step (pure Spurr) that was performed without any radiation. The polymerization step was carried out for 48 h in an oven at 70°C. The samples were sliced in 75 nm sections under a Leica EM UC7 ultramicrotome (Leica, Wetzlar, Germany), collected onto formvar-coated copper slot grids and submitted to incubation with 5% (w/v) uranyl acetate in water for 20 min and lead citrate for 5 min for contrasting. Finally, the samples were observed in a Tecnai^TM^ Spirit microscope operated at 120 kV (FEI Company, OR, United States).

### Electron Tomography and Three-Dimensional Reconstruction

The samples were processed following the same procedures described above [see Transmission Electron Microscopy (TEM)]. However, for electron tomography, a few different steps were performed, as follows. Samples were sliced into 200 nm thick serial sections under a Leica EM UC7 ultramicrotome (Leica, Wetzlar, Germany), collected onto formvar-coated copper slot grids and stained with 5% (w/v) uranyl acetate for 3 min and Reynolds’ lead citrate for 5 min. The tomographic series were acquired with an inclination of ±65° and 1° increment under a Tecnai Spirit^TM^ (FEI Company, Oregon, United States) transmission electron microscope operating at 120 kV with a 2,048 × 2,048 pixels’ matrix CCD camera. Serial tilt series were aligned using Etomo, open-source software from the IMOD package, a set of image processing, modeling and display programs used for tomographic reconstruction and three-dimensional reconstruction of EM serial sections (Kremer et al., 1996; Mastronarde, 1997). Generated tomograms were reconstructed using 3dmod.

### Statistical Analysis

Statistical analyses were performed using GraphPad Prism 8.4.3 (GraphPad Software, La Jolla, CA). Student’s *t*-test was used for comparisons.

## Results

### *Cryptococcus neoformans* Differentiates a Region of Its Cell Wall to Generate Daughter Cells

**FIGURE 1:**
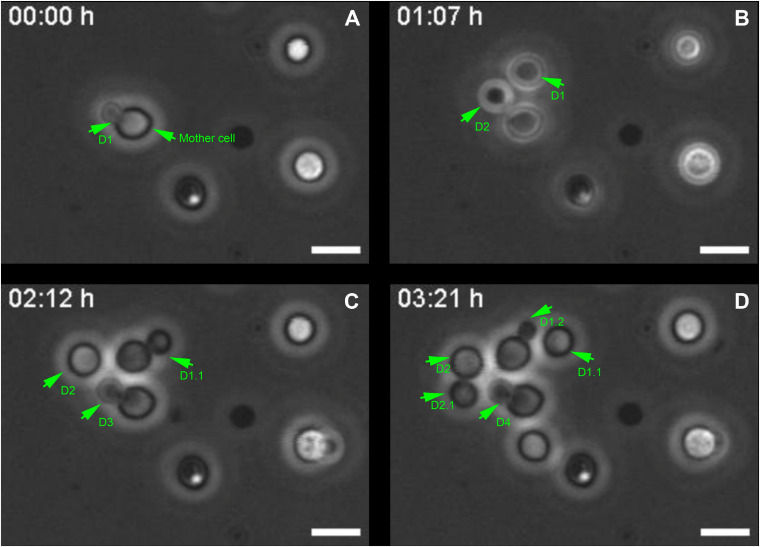
Selected images from a video microscopy movie of *Cryptococcus neoformans*, grown in Sabouraud medium, showing budding evolution. **(A–D)** Snapshots taken at 0, 1 h 07 min, 02 h 12 min, and 03 h 21 min, respectively, showing how the budding events evolve with time. Green arrowheads indicate yeasts during the division process that takes approximately (1.3 ± 0.3) h/cell. They also show that the daughter cells **(D)** always bud unipolarly and repeatedly from the same region of a mother cell. In the figure, D1 indicates first daughter cell, while D2, D3, and D4 all respectively indicate second, third and fourth daughter cells, all generated from the same mother cell at different time points. D1.1 indicates daughter cell from the second generation, the other numbers follow a similar logic. Scale bar is 5 μm. A total of 11 different budding events were evaluated. The result was expressed as mean ± SEM (Supplementary Video 1).

*Cryptococcus neoformans* divides through the budding of daughter cells from mother cells (Lin et al., 2014). To revisit and better characterize this phenomenon, yeast cell cultures were grown in Sabouraud Dextrose Broth medium and were allowed to attach onto coverslips. Next, we acquired phase-contrast images of the same field of view every minute for 5 h. Images were mounted in stacks allowing us to follow the proliferative behavior of the cells. Mother cells generated their daughters (D) at an average rate of one cell every (1.3 ± 0.3) h (Figure 1 and Supplementary Video 1). Moreover, successive daughter cells always bud from the same regions of their respective mothers, not only from cells that were attached since the beginning (Figures 1A–C and Supplementary Video 1), but also from daughter cells that subsequently attached to the coverslips after budding and also started to generate daughter cells of their own (Figures 1C,D and Supplementary Video 1), as previously observed by our group (Cordero et al., 2013).

Although this observation is already a consensus in the *Cryptococcus* field (Pierini and Doering, 2001; Zaragoza et al., 2006; Cordero et al., 2013) it led us to hypothesize that the mother cell might develop a specialization at the cell wall, creating a region with certain characteristics that might facilitate budding.

### *Cryptococcus neoformans* Mother Cell Wall Reorganizes Before the Budding of Daughter Cells

**FIGURE 2:**
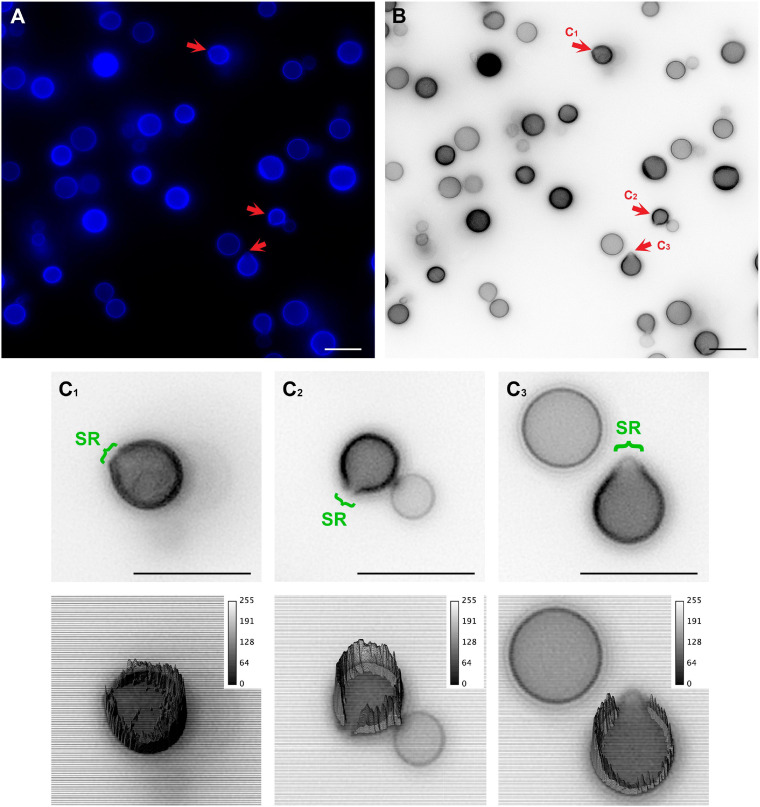
Conventional fluorescence microscopy images of *Cryptococcus neoformans* stained with Uvitex2D depicting chitin polymers at the cell wall. **(A)** Fixed and stained *C. neoformans* with chitin labeled in blue. Notice the discontinuity of the cell wall staining (red arrow). Those are the specialized regions (SR) in yeasts. **(B)** Gray scale image of panel **(A)**. Panels **(C1–C3)** are cells that are under different budding stages (see red arrows). **(C)** Zoom of panels **(C1–C3)** (upper panel) with their respective three-dimensional surface plots (lower panel). Surface plots indicate that the cell walls in SRs have a lower fluorescence intensity when compared to the entire cell wall rims. Scale bars are all 10 μm.

To unravel details of these specialized regions (SRs), *C. neoformans* yeast cell cultures were stained with Uvitex2B to label the chitin polymers present in the cell wall and subsequently were observed in a conventional fluorescence microscope. Images showed that mother cells formed regions of lower fluorescence intensities surrounding the SRs when compared to the rest of the cell perimeter (Figures 2A–C).

**FIGURE 3:**
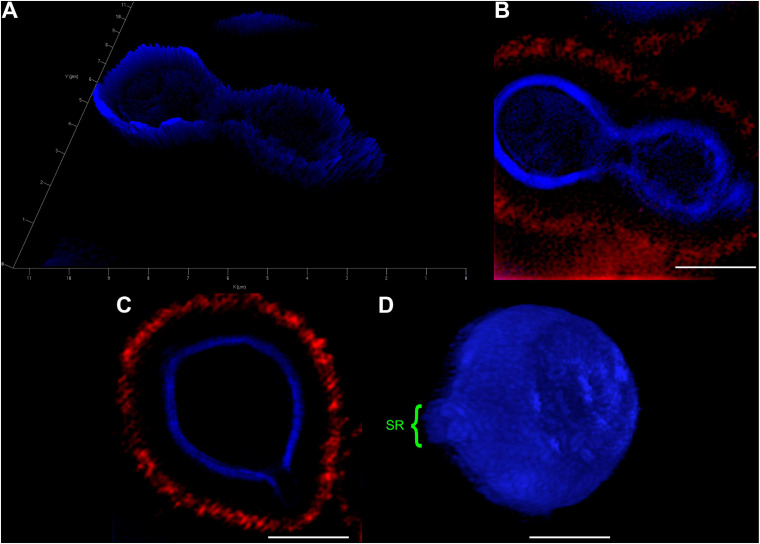
Structured Illumination Microscopy images of *Cryptococcus neoformans* confirm that both, the cell wall and capsule, deform prior to the budding event and generation of daughter cells. Yeast cells were fixed and stained for Uvitex2B (blue) and 18B7 + Alexa Fluor^®^ 594 antibody (red) so we could follow their walls and PS capsules, respectively, during budding and generation of daughter cells. **(A)** 2.5D yeast profile, showing that the mother cell (left) has a higher cell wall fluorescence intensity when compared to the daughter cell (right). **(B)** 2D representation of the same field as in panel **(A)**. **(C)** Representative image of a yeast cell at the beginning of the budding process. SR can be visualized as a discontinuity of the cell wall. **(D)** Three-dimensional profile of a representative yeast cell also in the beginning of the budding process showing the SR (highlighted in green). Scale bars: panel **(A)** 1 μm; panels **(B–D)** 2 μm (Supplementary Video 2).

In an attempt to better clarify the organization of these SRs, we also imaged *C. neoformans* cells using Structured Illumination Microscopy (SIM). Uvitex2B was used together with 18B7 + Alexa Fluor^®^ 594, to stain both the cell wall and the PS capsule respectively (Figure 3 and Supplementary Video 2). The results confirmed the decrease in fluorescence intensities around the SR when compared to the entire cell perimeter (Figures 3A–C). Furthermore, although with few details, it was possible to identify that the cell walls seemed to peel off to form the SRs from which daughter cells bud (Figure 3D).

### Ultrastructural Details of *C. neoformans* Specialized Regions During Budding

To better visualize the SRs with greater resolving power, we used TEM and three-dimensional reconstruction by electron microscopy.

**FIGURE 4:**
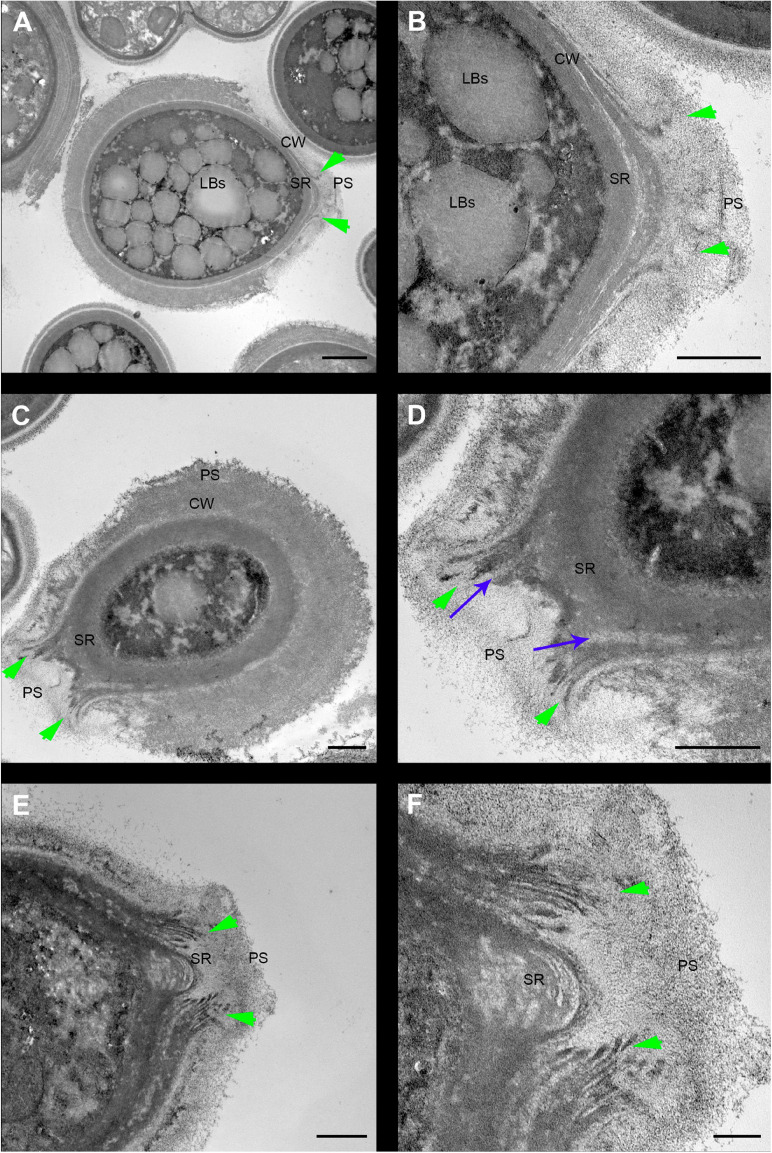
Transmission electron microscopy of *Cryptococcus neoformans* during budding reveals that both the cell wall and PS layer reorganize prior to budding. The right column images **(B,D,F)** are zoomed images of the panels represented on the left **(A,C,E)**. The green arrowheads indicate the cell wall delamination at different budding stages. **(A,B)** Yeast in the beginning of the budding process presents a modest cell wall delamination in the specialized region (SR). **(C,D)** Intermediate cell wall delamination stage and reduction of the PS fiber density at the SR when compared to the rest of the cell perimeter, in panel **(D)** blue arrows indicate, possibly, accumulation of chitin near the SR. **(E,F)** Discontinuity of the cell wall showing an advanced delamination step in which the cell wall peeling is evident and the reduction of the density and complexity of the PS fibers around the SR is evident. LBs: Lipid Bodies; CW: Cell Wall; PS: Polysaccharide; SR: Specialized region. Scale bars: panels **(A,C,E)** 1 μm; panels **(B,D,F)** 500 nm.

**FIGURE 5:**
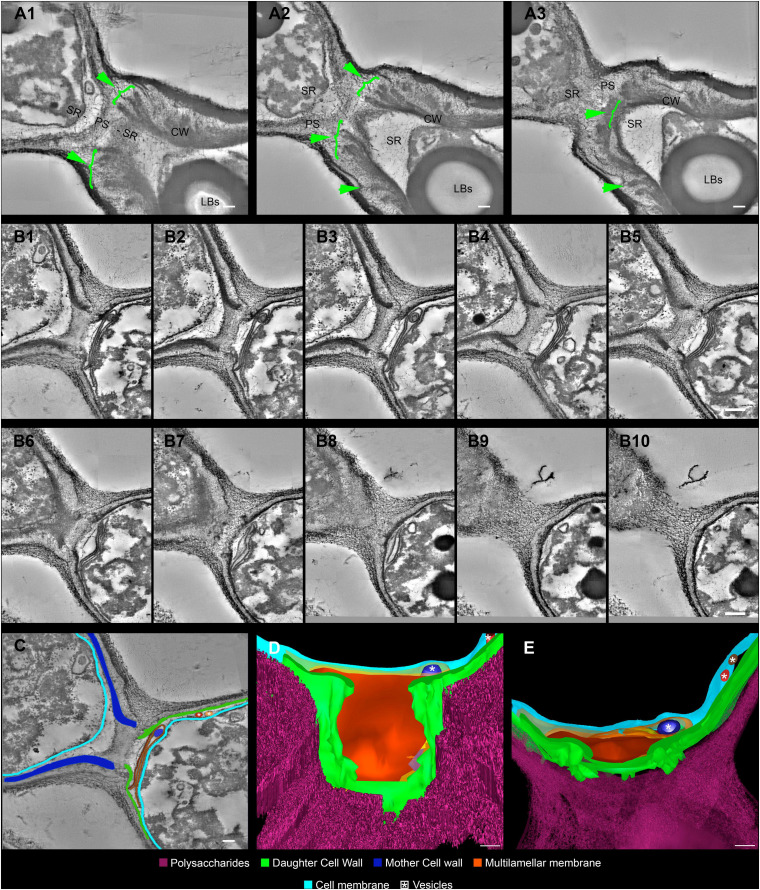
Serial electron tomography and three-dimensional reconstruction of *Cryptococcus neoformans* show ultrastructural details of the budding process. **(A)** Different *z*-planes of a serial tomogram of a budding event. The green arrowheads point to the cell wall delamination along different image planes. The mother cell is located at the bottom right, while daughter cell is at the top left. **(B_1__–__10_)** Set of sequential slices from another tomogram evidencing the modifications in cell wall cohesion along different angles. Note the presence of a multilamellar membrane and vesicles in synergism with the PSs surrounding the SRs, which probably acts as a shield. The top left cell is the mother cell, while the bottom right one is the daughter cell. **(C)** Segmentation of areas to be reconstructed three-dimensionally, highlighting the structures that participate and/or are remodeled in the SR. The top left cell is the mother cell while the bottom right one is the daughter cell. **(D,E)** Different views of the three-dimensional model from the daughter cell perspective. In panel **(E)**, we see a front view of the daughter cell wall discontinuity at the region of budding. This region is sealed by several membrane profiles as show in panel **(D)**. In panel **(E)**, a top view of the model where membrane profiles can be seen in between the CW and the cell membrane. Several vesicles (white asterisk) could be seen inside the membrane profiles (dark blue) and also between CW and cell membrane (red and brown). LBs: Lipid Bodies; CW: Cell Wall; PS: Polysaccharide; SR: Specialized region. Scale bars are all 100 nm (Supplementary Videos 3, 4).

*Cryptococcus neoformans* yeast cell cultures were imaged using TEM. By using this technique, we were able to observe the budding process in detail (Figures 4, 5). Our observations demonstrated that the process started with the shape change of one of the poles of the cell which deformed, lost sphericity and formed a more pointed region in the cell wall that, at some point, began to break into layers (Figures 4A,B). As the daughter cell started to appear, the formed layers became more evident with thick and electron-dense tips slightly separated from each other (Figures 4C,D). When the daughter cell is released, a new budding event is likely to arise in the same region (Figures 4E,F).

For the first time, electron tomography and three-dimensional reconstruction of *Cryptococcus* cell wall were also performed, allowing us to observe further details of the budding events (Figure 5 and Supplementary Videos 3, 4). Strikingly, with the tomography series, it became clearer that not only is there a separation of the mother cell wall into layers with thick tips (Figures 5A–C), as previously described (Figure 4), but also that the mother cell wall is thicker than the daughter cell wall (Figures 5B,C). Interestingly, daughter cells presented multilamellar membranous structures covering the continuous openings between daughter and mother cells (Figures 5B–E). It also became evident that the entire budding process induced capsule reorganization around the SRs (Figures 4, 5). The most striking change observed in capsule morphology during budding was the formation of a protective PS barrier surrounding the SRs of both mother and daughter cells (Figure 5). Three-dimensional reconstruction demonstrated all of these characteristics (Figures 5D,E and Supplementary Video 4).

### Capsule PS Fibers Around Specialized Regions of Daughter Cells Are Oriented Toward the Budding Events

**FIGURE 6:**
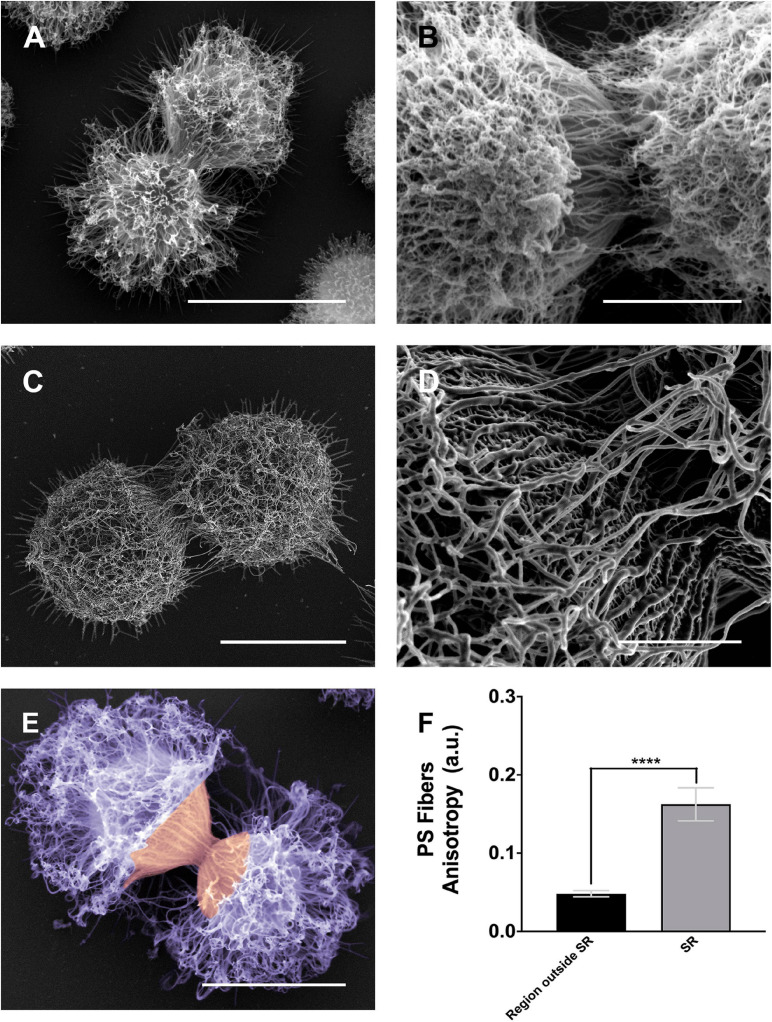
Scanning electron microscopy images of *Cryptococcus neoformans* showing the alignment of the PS fibers near the budding region. **(A,B)** Different examples showing changes in the spatial and conformational orientation of both mother and daughter PS capsules near the budding region. Panel **(A)** representative image of a budding event viewed by conventional scanning electron microscopy; the microscope used to obtain this specific image was a FEI Quanta^TM^ 250 operating at 20 kV. Panel **(B)** representative image of a budding event viewed by high-resolution scanning electron microscopy, the microscope used to obtain this image was a FEI Quanta 450 FEG operating at 5 kV. Panels **(C,D)** representative image of another budding event viewed by high-resolution scanning electron microscopy panel **(C)** and its zoom panel **(D)** highlighting the PS fiber alignment, the microscope used to obtain these images was a FEI Magellan^TM^ operating at 1 kV. Panel **(E)** another budding event viewed by conventional scanning electron microscopy; the microscope used to obtain this specific image was a FEI Quanta 250 operating at 15 kV. In purple, the dispersed PS fibers located at the region outside SR, while in orange the aligned PS fibers in the SR. **(F)** Plot of the mean anisotropy values, arbitrary units (a.u) of PS fibers around SRs (gray) and regions outside SRs (black). Twenty-three different measurements were performed for each experimental situation. Standard errors were used as error bars. ****p < 0.0001 in Student’s *t*-test statistics. Scale bars for panels **(A,C,E)** are 5 μm and for panels **(B,D)** are 2 μm.

To clarify the details of capsule remodeling around the SRs of both mother and daughter cells, *C. neoformans* were processed and visualized by conventional and high-resolution scanning electron microscopes (Figure 6).

As previously described (Figures 4, 5), the images presented in Figure 6 confirmed the PS remodeling around the SRs, but now adding more details to the rearrangement. The PS fibers surrounding the SRs from both daughter and mother cells showed a distinct spatial organization. While daughter cells presented fibers aligned in the direction of budding, mother cells exhibited fibers similarly aligned but in an opposite direction (Figures 6A–C). To better quantify these visual observations, we performed a quantitative analysis using the FibrilTool plug-in (Boudaoud et al., 2014). The results (Figure 6D) showed that, overall, the PS fibers around SRs present a higher anisotropy when compared to fibers outside the SRs. This special arrangement around SRs may be related to a mechanical protruding force that occurs during budding.

## Discussion

Cellular events during *C. neoformans* yeast cell morphogenesis are well described, but still lack information regarding ultrastructural modifications. In general, cells undergo a cell division cycle with asexual and repeated clonal budding of a haploid yeast cell; however, unlike *S. cerevisiae*, in which subsequent budding events occur adjacent to previous scars, *C. neoformans* preferentially and repeatedly generates their daughter cells from the same location, as previously demonstrated (Pierini and Doering, 2001; Zaragoza et al., 2006; Cordero et al., 2013) and also as shown in the present study.

Cell division in *C. neoformans* is a highly dynamic mechanism influenced by environmental conditions. The analysis of cell duplication time in different nutritional environments shows a huge mean variance of 1.29 ± 0.03 h in the generation time of daughter cells. In previous work from our group, *C. neoformans* cells were placed in an environment with nutritional deprivation and the average doubling time found was 2.1 ± 0.2 h (Cordero et al., 2013). The variability in doubling time was also visible individually between cells of the same condition, with a mean variance of 0.31 ± 0.04 h (Cordero et al., 2013). However, in the present study, a nutrient-rich environment, with accessibility to abundant carbon sources, was used and we demonstrated that *C. neoformans* divide with shorter generation times (1.3 ± 0.3 h).

To unravel details of *C. neoformans* cell wall remodeling, we stained yeast cell cultures with Uvitex2B to label the chitin polymers present in the cell wall (Koch and Pimsler, 1987). Our results show that mother cells form regions of lower fluorescence intensities surrounding the SRs when compared to the rest of the cell perimeter. A similar experiment was carried out by Pierini and Doering (2001), although they used the 40-(aminomethyl) fluorescein to stain the cell wall. The weak fluorescence staining surrounding the budding region or even the budding cell suggests that mother cell wall content is not transferred to the wall of the newly formed daughter. Thus, supporting the hypothesis that the cell wall rearrangement may be related to the formation of some sort of rupture, opening or even partial displacement of the mother cell wall so that the new daughter can be formed during budding. Our results not only corroborate this hypothesis but also describe the morphological features of how this remodeling occurs in *C. neoformans*. We show that the cell wall starts to form a fissure-like structure (Figures 4A,B) that culminates with the opening of a specialized region (Figures 4C–F) where *C. neoformans* preferentially and repeatedly generates its daughter cells, as previously stated (Pierini and Doering, 2001; Zaragoza et al., 2006; Cordero et al., 2013). The cell wall is then reorganized into layers around the SRs. Similar to what was documented more than 30 years ago (Lurie et al., 1971; Baharaeen and Vishniac, 1981) for other yeast species, but quite neglected since that time. We conjecture that the formation of these specialized regions, originated from the opening of mother cell walls and showing separated cell wall layers with thick tips delimiting the region, is related to a mechanical protruding force coming from inside the mother cell during budding. Besides being essential for budding, the presence of these SRs could be used, for example, as outlets for extracellular vesicles, whose mechanisms of passage through the cell wall are still unclear (de Oliveira et al., 2020). Our results show extracellular vesicles in the periplasmic space surrounding the SRs (Figure 5D). We speculate that the SRs could be more prone to the passage of vesicles, however further studies are needed to confirm this hypothesis. Apart from the mechanical aspect, we cannot completely rule out the possibility of a change in biochemical composition to facilitate this process. It is known that the cell wall is formed by specific components. The inner part is mainly composed of β-glucan and chitin arranged as fibers parallel to the plasma membrane and the outer part contains α-glucan and β-glucan (Sakaguchi et al., 1993; O’Meara and Alspaugh, 2012). However, it has been shown that chitin accumulates as a ring surrounding the budding scar in *S. cerevisiae* yeasts. Moreover, cross-sectional images seen in a transmission electron microscope highlight the electron-lucent regions around the bud scar as chitin accumulation (Cabib and Bowers, 1971). Similarly, our TEM images point to electron-transparent regions (see blue arrows of Figure 4D). We speculate that these regions could also be chitin accumulation around the SRs of *C. neoformans* and suggesting that chitin may play a crucial role in these regions of the cell wall after their remodeling for budding events. Moreover, several cell wall proteins have been described to have key roles in capsule architecture. Some of these proteins, such as the GPI-linked β-glucanase Gas1, have been implicated in remodeling of the cell wall as it directly acts on β-1,3-glucans (Levitz and Specht, 2006; Eigenheer et al., 2007). Therefore, preceding cell budding, the structural components of the cell wall may be reorganized to form a region where budding is facilitated. In the present study, we describe the morphological aspects of the cell wall remodeling; however, other specific molecular and mechanical details constitute lines of investigation for future studies.

In order to avoid introducing artifacts on the surface of *C. neoformans* during TEM processing, we decided to perform various tests with distinct protocols. After several attempts, the protocol that best preserved the structures was an adaptation of the OTO protocol, using shorter steps and introducing microwave electromagnetic stimuli (for more details, see the materials and methods section). We found that longer incubation times at each step resulted in extremely electron-dense samples, which considerably reduced the visualization of SR details. The use of microwave pulses had a great advantage in reducing the fixation, post-fixation and resin polymerization times, mainly because the electromagnetic waves promote an increase in kinetic energy, favoring the diffusion of fixatives and other fluids and thus producing better preservation of the material, as previously described (Seligman et al., 1966; Willingham and Rutherford, 1984; Benchimol et al., 1993; Giberson et al., 2003; Wendt et al., 2004; Tapia et al., 2012). Moreover, there are at least five different cytochemical methods used to identify carbohydrates: (i) based on the fact that the carbohydrates have vicinal hydroxyls susceptible to oxidation by periodic acids; (ii) based on the acidic nature of some carbohydrates such as uronic acid and acid derivatives neuramine (alcian blue, ruthenium red, cationized ferritin, colloidal iron, poly-lysine associated with colloidal gold particles, etc.); (iii) based on lectins; (iv) based on the use of specific enzymes with subsequent visualization of their binding sites; and (v) based on immunocytochemistry methods using monoclonal or polyclonal antibodies. In the present work, we decided to give a more global view of the ultrastructural changes that occur on the surface of *C. neoformans* during budding events. Further observations correlating the biochemical details with morphological changes that occur in these specialized regions can be explored in future studies.

Beyond the cell wall and attached to its surface lies the PS capsule. Despite its homogeneous appearance, when viewed through light microscopy, several lines of evidence show that the capsule is a highly heterogeneous structure with a complex and dynamic spatial organization. It is known, for example, that the capsule matrix exhibits clear vertical stratification, with distinct density regions, with its inner part having a higher fiber density than its outer region (Gates et al., 2004; Bryan et al., 2005; Frases et al., 2009b; Araújo et al., 2016, 2017). Although softer than the cell wall and presenting viscoelastic properties (Frases et al., 2009a; Araújo et al., 2019), the high PS density of the inner region prevents the penetration of larger macromolecules, including antibodies and proteins of the complement system, restricting the access of these molecules to the cell wall (Gates et al., 2004; Gates and Kozel, 2006). Therefore, one could speculate that the capsule might also undergo remodeling during budding. Indeed, our results show that the PSs constituting the capsule experience shape changes around the SRs of both mother and daughter cells (Figure 6). Similar images were previously documented by Zaragoza et al. (2006 – Figure 9A of their study) and a model, based on the formation of an initial dimple that subsequently forms a tunnel/hole in the capsule of the mother cell around the budding region (Zaragoza et al., 2006 – Figure 10C of their study), was presented. Although these authors did not explain how the tunnel/hole is formed, they conjectured that it could facilitate the passage of daughter cells. However, in their model, the mechanical aspects related to the budding of the daughter cell was never mentioned. In a subsequent study, trying to observe whether the presence of different antibodies against the PSs could interfere in the elastic properties of the capsule and, as a consequence, in the dynamics of the budding process, our group performed video microscopy experiments with *C. neoformans* cells previously treated with non-protective 13F1 IgM antibodies [(Cordero et al., 2013) – Supplementary Video 3^2^]. Yeast cells previously treated with 13F1 showed a discrete halo around the capsule edge. Although quite discrete, it allows to observe the capsule rearrangement during budding. As the daughter cell grows, it pushes and displaces the capsule of the mother cell, facilitating its passage (Cordero et al., 2013). All previously mentioned findings, together with the results of the present study, enable us to conjecture that the capsule rearrangement is due to a mechanical protruding force occurring inside the mother cell during budding. The change in PS shape seems to be correlated with this conjectured protruding force, as capsule fibers of daughter cells tend to orient toward the budding event, while the fibers from mother cells tend to orient in the opposite direction (Figure 6), similar to what happens during the launch of a projectile or rocket. Although these mechanical protruding forces have not yet been measured, it is believed to be within the same order of magnitude as those of other cellular events (in the pN range – 10^–12^ N) (Pontes and Frases, 2015), which makes optical tweezers the ideal instrument for their precise determination.

**FIGURE 7:**
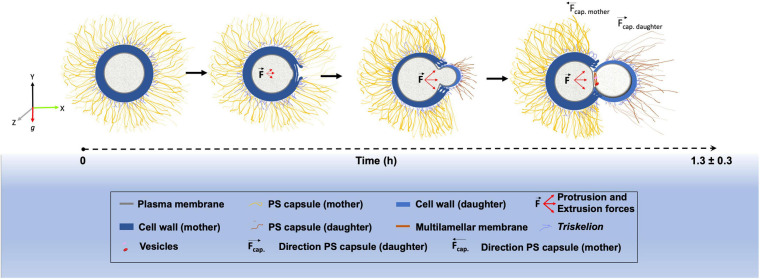
Schematic representation showing the morphological alterations that dynamically occur at the surface of *Cryptococcus neoformans* during budding.

Finally, we outlined a model highlighting the main morphological changes that occur within the cell wall, PS capsule and plasma membrane during the budding process in *C. neoformans* (Figure 7).

## Conclusion

In conclusion, we have combined sophisticated light and electron microscopy techniques to describe the structural changes that occur between the capsule, cell wall and plasma membrane during the budding phenomenon in *C. neoformans*. We have presented, for the first time, three-dimensional reconstruction serial tomography experiments supporting a possible remodeling through mechanical protruding forces originated inside the yeast mother cell, although the magnitude of such force and the mechanisms behind its generation have yet to be elucidated. All morphological changes observed create a specialized region with characteristics that seem to favor budding events, which can partially explain why budding in *C. neoformans* always occur in the same region. However, the complete mechanism may also involve a controlled rearrangement of the molecules that constitute both the cell wall and the PS capsule. Such remodeling would probably affect not only the biochemical but also the mechanical features of both structures, probably changing capsule stiffness in the budding region. All these modifications could be explored in future studies. We also aim to explore and characterize further these specialized regions such that they could be used as potential drug-targets against cryptococcosis.

## Data Availability Statement

The original contributions presented in the study are included in the article/Supplementary material, further inquiries can be directed to the corresponding authors.

## Author Contributions

GA contributed to conceptualization, methodology, investigation, visualization, data curation, formal analysis, writing – original draft, and writing – review and editing. CA and NR contributed to methodology and writing – review and editing. WS contributed to supervision, funding acquisition, formal analysis, and writing – review and editing. BP and SF contributed to conceptualization, methodology, investigation, visualization, data curation, resources, supervision, funding acquisition, formal analysis, writing – original draft, and writing – review and editing. All authors contributed to the article and approved the submitted version.

## Conflict of Interest

The authors declare that the research was conducted in the absence of any commercial or financial relationships that could be construed as a potential conflict of interest.

## Footnotes

1 https://imagej.nih.gov/ij/

2 https://www.jimmunol.org/highwire/filestream/326166/field_highwire_adjunct_files/5/Movie_S3.mov

## Supplementary Material

Supplementary Video 1 Budding phenomena observed in a representative *C. neoformans* cell culture (related to Figure 1).

Supplementary Video 2 Structured illumination microscopy (SIM) reconstruction of a representative *C. neoformans* cell in the beginning of the budding process showing the SR (related to Figure 3D).

Supplementary Video 3 Set of electron tomography planes showing *C. neoformans* mother and daughter cells during a budding process (related to Figure 5A).

Supplementary Video 4 Set of electron tomography planes showing *C. neoformans* mother and daughter cells during a budding process together with a 3D model from serial images acquired by serial-section transmission electron microscopy of *C. neoformans*, where it is possible to evidence the specialized region (SR). In purple polysaccharides, in green cell wall of the daughter cell, in blue cell wall of the mother, in orange multilamellar membrane and in cyan cell membranes (related to Figures 5B–E).
